# Latent profile analysis on the decent work of Chinese employees: applying the Psychology of Working theory

**DOI:** 10.3389/fpubh.2024.1364431

**Published:** 2024-08-29

**Authors:** Shengnan Wang, Kai Feng, Fengyuan Chu, Yongxin Li, Guoxiang Zhao

**Affiliations:** ^1^Mental Health Service Center, Henan University of Animal Husbandry and Economy, Zhengzhou, China; ^2^Institute of Psychology and Behaviour, Henan University, Kaifeng, China; ^3^Academic Affairs Office, Henan University of Animal Husbandry and Economy, Zhengzhou, China; ^4^College of Culture and Social Sciences, Chonnam National University, Yeosu, Republic of Korea

**Keywords:** decent work, Chinese employees, Psychology of Working theory, work need satisfaction, job satisfaction, life well-being

## Abstract

**Background:**

This study used a person-centered approach to identify the specific performance of decent work in various groups to determine the heterogeneity of its five dimensions.

**Method:**

The Decent Work Scale, Work Need Satisfaction Scale, Socioeconomic Status Scale, Job Satisfaction Scale, and Life Well-being Scale were used to conduct a network survey of organizations in various industries in Mainland China. A total of 1,000 questionnaires were distributed, and 780 valid responses were obtained.

**Results:**

The results showed that the decent work of participants could be divided into three types: low salary, low free time, and high decent work. The results showed no significant difference in age among the groups, whereas the differences in socioeconomic status were significant. Welch’s test was used to determine differences in the positive outcomes of the three potential types of decent work, and the results showed significant differences in work need satisfaction, job satisfaction, and life well-being among all groups.

**Conclusion:**

This study examined the characteristics of decent work more realistically, showing that decent work is not an all-or-nothing structure and that its intrinsic components should be flexibly combined according to the research background and purpose.

## 1 Introduction

The Psychology of Working theory suggests that decent work is a crucial lever for fulfilling individual psychological needs, enhancing well-being, and promoting dignity and sustainable development in society (1). Nonetheless, perceptions of decent work are shaped by employees’ specific experiences at the workplace. Research indicates that decent work is a multidimensional construct comprising several dimensions (1, 2). The Psychology of Working theory measures decent work by examining employees’ evaluations of five specific aspects of their work: a safe work environment (safety dimension), access to healthcare (healthcare dimension), appropriate compensation (compensation dimension), reasonable working hours (free time dimension), and organizational values aligned with family and social values (value fit dimension) (1).

Recent international studies on decent work have yielded diverse findings across cultural contexts. For instance, research in the United States by Blustein et al. (3) and Kim et al. (4) identified five latent profiles of decent work, with healthcare being a prominent indicator of profile segmentation. In contrast, studies in European contexts have emphasized the importance of work-life balance and job security in defining decent work [e.g., (2)]. Our study contributes to this growing body of literature by examining decent work profiles in the Chinese context, revealing unique patterns that both align with and diverge from previous international findings.

The concept of decent work has been subject to various interpretations and critiques across disciplines. While the International Labour Organization (ILO) defines decent work in terms of employment opportunities, rights at work, social protection, and social dialogue (5), scholars have expanded and challenged this definition. For instance, Deranty and MacMillan (6) argue for the inclusion of the qualitative experience of work, emphasizing the importance of meaningful work. From a capabilities approach, Sehnbruch et al. (7) propose that decent work should be viewed as a multidimensional concept that encompasses not only employment conditions but also individual freedoms and opportunities for personal development. Critics like Standing (8) contend that the decent work agenda may be too broad and difficult to operationalize effectively. Our study contributes to this ongoing debate by examining how different dimensions of decent work manifest in the Chinese context, providing empirical evidence for the multidimensional nature of the concept.

These five dimensions of decent work may exhibit different levels of heterogeneity in actual work situations. Individuals’ perceptions of work are based on their categorical cognitive schemas, which are formed through a combination of various attributes and characteristics (9). Therefore, some jobs widely recognized as decent may not possess all five dimensions. For instance, some freelancers with knowledge-based or skilled work may have a high income, free time, and enjoyable work but lack adequate healthcare and social security benefits. Conversely, some jobs may have complete medical insurance, but require high-risk work environments. Others may have high income, a good environment, and complete medical insurance but require long working hours and lack sufficient rest time. In summary, different types of work offer distinct pathways to the various dimensions of decent work, highlighting that it is not an all-or-nothing concept.

Blustein (10) further nuances the concept of decent work by distinguishing between “decent work” and “decent work with meaning.” He argues that while stability often makes work decent, it does not necessarily make it meaningful. This distinction is particularly relevant in the Chinese context, where rapid economic development has led to increased job stability in many sectors, but questions remain about the meaningfulness of work for many employees. Our study explores this nuance by examining not only the presence of decent work characteristics but also their relationship with work need satisfaction and well-being, providing insights into the potential meaningfulness of work across different profiles.

The concept of decent work holds particular relevance in the Chinese context, given the country’s rapid economic transformation and evolving labor market. According to the National Bureau of Statistics of China (11), the urban unemployment rate in China stood at 5.2% in 2020, with significant variations across regions and industries. The Chinese government has implemented various initiatives to promote decent work, including the “Employment First” strategy and improvements in social security coverage (12). However, challenges persist, such as income inequality, with a Gini coefficient of 0.465 in 2019 (13), and concerns about work-life balance in urban areas (14). Our study contributes to understanding how these broader socioeconomic factors manifest in employees’ experiences of decent work.

The Decent Work Scale (DWS) (15) is a useful tool for comprehensively measuring the five dimensions of decent work. Many studies have used the DWS to obtain composite scores for decent work and explore its relationships with various predictors and consequences. However, the variable-centered approach, which treats decent work as a unified structure, has certain limitations. Although this approach is concise and efficient, it assumes sample homogeneity and masks heterogeneity among the five dimensions of decent work. This assumption impedes our ability to examine the specific characteristics or performance of decent work across different groups and gain insight into the predictors and outcomes of decent work across various characteristics. In recent years, some scholars have called for a more person-centered approach that accounts for heterogeneity in the five dimensions of decent work (4).

Kim et al. (4) measured the characteristics of decent work of 2,465 American adults through random sampling and processed the data using latent profile analysis (LPA). The results showed that the characteristics could be divided into five latent types: common (scores close to the theoretical mean), low healthcare security, less decent, high healthcare security, and decent. These five potential types differed significantly in terms of demographic variables (such as gender and educational level), predictors within the Psychology of Working theory framework (such as economic constraints, marginalization, and career determination), and outcome variables (such as job and life satisfaction).

Both the aforementioned studies were conducted in the context of the United States and classified subjects into five potential types, with the healthcare dimension being a prominent indicator of the segmentation profile. However, China and the United States have significant differences in their social systems, history, and culture. Therefore, the research results obtained in the American context may not be entirely applicable to China. For example, China’s medical security system is significantly different from that of the United States, with China’s system being dominated by social medical insurance and that of the United States’ being dominated by commercial medical insurance (16). In terms of culture, Hofstede’s (17) research on the national cultural dimension indicates that China is representative of an Oriental culture, is a highly collectivist country, and attaches considerable importance to individuals’ social responsibility. Conversely, the United States is representative of the Western culture, is a highly individualistic country, and emphasizes personal material achievement. Studies have shown that differences in social systems and cultures shape different job characteristics, affecting individuals’ work behaviors, attitudes, and the formation of values (18), which, in turn, affect their performance and work feelings (19). Thus, Chinese employees’ decent work may have unique characteristics. Based on this, we adopted a person-centered research approach, which involves classifying individuals into subgroups based on shared response patterns, in an attempt to capture observed heterogeneity in decent work experiences (20, 21). This study identified differences in five dimensions of decent work by examining its specific performance in various groups, identifying the heterogeneity of its five dimensions, and providing a concrete description of decent work closer to real-life experiences in the Chinese context. This approach will help probe the underlying subtle makeup of decent work and reveal the convergence and divergence between its components by identifying the characteristics of decent work in different subgroups.

## 2 Methods

LPA is an emerging person-centered analysis technique which is part of the broader family of mixture models that assume a normal distribution within each latent class. This statistical method explains the relationship between explicit continuous indicators through latent categorical variables. Objective statistical indicators are used to measure the accuracy and validity of classification, maximize homogeneity between heterogeneous groups, and effectively identify the unique characteristics of individual psychological behavior (21, 22). The underlying assumption is that the probability distribution of various responses to an explicit variable can be explained by a few mutually exclusive latent class variables, each with a specific preference for explicit variable responses (23, 24). Similar to traditional mean segmentation and cluster analysis, the LPA method is individual-centered, treats the variables in the study as an interdependent system, and divides the sample into multiple subgroups (latent types). Further, it analyses the antecedents and after-effects of subgroups’ characteristics to achieve a more intuitive and realistic description of the subjects and improve understanding. However, compared with traditional mean segmentation, cluster analysis, and other techniques, LPA makes classifications based on model fitting estimation and does not require high data dimensions, significant causal relationships between variables, or large sample data from multiple time points. Usually, the sample size is greater than 500, making the classification more accurate and objective and the operation simpler (21, 25).

In the data analysis process, the potential profile analysis method comprehensively refers to three types of indicators—information, likelihood ratio test, and classification indicators—to judge the model’s goodness of fit. Information indicators include the Akaike information criterion (AIC), Bayesian information criterion (BIC), adjusted Bayesian information criterion (aBIC), and bootstrap likelihood ratio test (BLRT). The classification index mainly refers to entropy. Under normal circumstances, the smaller the AIC, BIC, and aBIC values, the higher the model’s goodness of fit. The higher the entropy value, the higher the model’s classification accuracy. If the *p*-value of the BLRT is less than 0.05, this indicates that the category model’s goodness of fit is significantly improved compared with that of the previous model. Additionally, theoretical factors and previous research results should be considered comprehensively when determining the number of profiles. Each profile obtained should be logically self-consistent and provide a reasonable theoretical explanation. A relatively parsimonious model is typically used when profiles have similar theoretical characteristics (21).

### 2.1 Participants

This study adopted random sampling and used the Questionnaire Star platform to conduct online testing in enterprises and institutions across various industries in Mainland China between March and July 2021. A total of 1,000 questionnaires were distributed; 889 were returned, and 780 valid questionnaires remained, resulting in a response rate of 78%. The samples were distributed across all provinces and municipalities in the country. Among them, 107 were from private enterprises, 252 were from state-owned enterprises, 365 were from government agencies or institutions, and 56 were self-employed or from other professional backgrounds. The sample comprised 318 male participants (40.8%) and 462 female participants (59.2%). The age range of the participants was 21–60 years (mean; *M* = 31.638, standard deviation; *SD* = 5.9657), and their working age ranged from 1 to 35 years (*M* = 7.38, *SD* = 5.633). Participants reported working 10–70 h per week (*M* = 43.41, *SD* = 8.591). Regarding educational attainment, 6 participants (0.8%) reported having a high school education or below, 16 (2.1%) had a college education, 463 (59.4%) reported having a bachelor’s degree, 235 (30.1%) had a master’s degree, and 60 (7.7%) reported having a doctoral degree.

### 2.2 Measurements

#### 2.2.1 Decent work

The DWS, developed by Duffy et al. (15), was used to measure decent work. The scale comprises five dimensions: safe environment, medical security, appropriate income, free time, and value fit. The safety environment dimension has three questions (e.g., ‘I feel emotionally secure when interacting with people at work’). The medical security dimension has three questions (e.g., ‘I can enjoy good medical insurance provided by the country and the government’). The appropriate income dimension has three questions (e.g., ‘I get the salary I deserve for my work’). The free time dimension has three questions (e.g., ‘I have sufficient personal time on working days’). The value fit dimension has three questions (e.g., ‘The values advocated by my unit are consistent with those of my family’). A total of 15 questions were asked. The scale uses a seven-point Likert scoring method, ranging from ‘1 = strongly disagree’ to ‘7 = strongly agree,’ with a minimum score of 15 points and a maximum score of 105 points. Higher scores indicated that the participants felt that their jobs met the characteristics of decent work. In this study, Cronbach’s alpha for this scale was 0.834.

#### 2.2.2 Work need satisfaction

The Work Need Satisfaction Scale (WNSS), prepared by Autin et al. (26), was translated into Chinese using the translation-retranslation method. This scale comprises five dimensions: survival needs, social contribution, job competency, interpersonal connections, and work autonomy. There are four questions for the survival needs dimension (e.g., ‘The income from work is sufficient to cover the living expenses of my family’), four questions for the social contribution dimension (e.g., ‘Work contributes to the sustainable development of society and makes me feel my value’), four questions for the job competency dimension (e.g., ‘I can complete my work tasks well’), four questions for the interpersonal connection dimension (e.g., ‘I can experience the feeling of being understood at work’), and four questions for the work autonomy dimension (e.g., ‘I can independently choose the way to complete work tasks’), comprising a total of 20 questions. The scale uses a seven-point Likert scoring method, ranging from ‘1 = strongly disagree’ to ‘7 = strongly agree,’ with a minimum score of 20 points and a maximum score of 140 points. The higher the score, the more the participants thought their work could meet their psychological needs. In this study, Mplus 8.3 was used to analyze the data, and the reliability analysis results showed that the overall Cronbach’s Alpha of the Chinese version of the WNSS was 0.868. Cronbach’s alphas of the subsistence needs, social contribution, job competency, interpersonal connection, and work autonomy subscales were 0.763, 0.692, 0.594, 0.689, and 0.804, respectively. The results of the confirmatory factor analysis showed the following: 2/df = 5.608, standardized root mean square residual (SRMR) = 0.073, Tucker-Lewis index (TLI) = 0.925, comparative fit index (CFI) = 0.935, and root mean square error of approximation (RMSEA) = 0.077. The factor loading of each item was above 0.5. The results showed that the Chinese version of the WNSS had good reliability and validity and that the five-dimensional structure of work need satisfaction was suitable for Chinese employees.

#### 2.2.3 Socioeconomic status

Participants’ subjective socioeconomic status was measured using Adler’s MacArthur ladder scale (27). Participants were shown a picture of a 10-level ladder and were asked to imagine that the ladder represented their social status. They were asked to rate their social status by indicating where they stood on the ladder on a 10-point scale ranging from 1 (lowest social status) to 10 (highest social status). At the top of the ladder (10) are those with the highest socioeconomic status—those who have the most assets, best education, and best jobs. At the bottom of the ladder (1) are those with the lowest socioeconomic status—those with the least assets, worst education, and no jobs.

#### 2.2.4 Job satisfaction

This was measured using the Job Satisfaction Scale developed by Tsui et al. (28), which contains six items, such as ‘I am very satisfied with the promotion opportunities in my unit,’ ‘I am very satisfied with the work I am doing,’ and ‘Generally speaking, I am very satisfied with my current job.’ The scale uses a five-point Likert scoring method, ranging from ‘1 = strongly disagree’ to ‘5 = strongly agree,’ with a minimum score of 6 points and maximum score of 30 points. The higher the score, the higher the participant’s job satisfaction. In this study, Cronbach’s alpha for the scale was 0.801.

#### 2.2.5 Life well-being

The life well-being subscale of the employee well-being scale compiled by Zheng et al. (29) was used to measure the participants’ well-being. The scale contains six items such as ‘Most aspects of my life are close to my ideals,’ ‘I am satisfied with my life,’ and ‘If there is an afterlife, I would hardly change my current lifestyle.’ The scale uses a seven-point Likert scoring method, ranging from ‘1 = strongly disagree’ to ‘7 = strongly agree,’ with a minimum score of 7 points and maximum score of 42 points. The higher the score, the better the participants believed they were living. In this study, Cronbach’s alpha for this scale was 0.851.

The two primary measurement instruments used in this study—the Decent Work Scale (DWS) and the Work Need Satisfaction Scale (WNSS)—are theoretically compatible and share a common epistemological basis. Both scales were developed within the framework of the Psychology of Working Theory (PWT) (1), which posits that decent work is a key mechanism for fulfilling basic psychological needs and promoting well-being.

The DWS, developed by Duffy et al. (15), operationalizes the five dimensions of decent work as conceptualized in the PWT. Similarly, the WNSS, created by Autin et al. (26), measures the extent to which work satisfies basic psychological needs, which is a central tenet of the PWT. Both scales were constructed using rigorous psychometric procedures, including exploratory and confirmatory factor analyses, and have demonstrated good reliability and validity across various cultural contexts.

Moreover, both instruments are grounded in self-determination theory (30), which emphasizes the importance of basic psychological need satisfaction for optimal functioning and well-being. This shared theoretical foundation ensures that the constructs measured by these instruments are conceptually aligned and can be meaningfully integrated within the same research framework.

### 2.3 Data analysis

We employed a multi-step analytical approach using Mplus version 8.3 (31) and SPSS version 26.0 (32).

First, we conducted descriptive analyses and calculated Cronbach’s alpha coefficients to assess the reliability of our measures. Second, we performed a Latent Profile Analysis (LPA) to identify distinct profiles of decent work. We tested models with one to four profiles, using multiple indicators to determine the optimal number of profiles. Third, we conducted a series of analyses to examine differences among the identified profiles:

- For categorical variables (gender, marital status, education, partner education), we used chi-square tests of independence.
- For continuous variables (age, socioeconomic status), we used one-way ANOVAs. When the assumption of homogeneity of variances was violated (as indicated by Levene’s test), we used Welch’s ANOVA followed by Games-Howell post-hoc tests.
- For outcome variables (work need satisfaction, job satisfaction, life well-being), we again used one-way ANOVAs or Welch’s ANOVA as appropriate, followed by post-hoc tests.

Finally, we calculated effect sizes (Cramer’s V for chi-square tests, *η*^2^ for ANOVAs) to assess the magnitude of differences among profiles.

## 3 Results

### 3.1 Analysis results of latent profiles

According to the fitting index table of the LPA model (Table 1), as the number of profiles increased from one to four, the AIC, BIC, and aBIC gradually decreased, indicating that the model’s fitting degree improved. However, from the three-section model to the four-section model, the entropy decreased significantly. Therefore, we concluded that the three-section model provides a better fit.

**Table 1:** Fitting index of the latent profile analysis model.

Number of sections	AIC	BIC	aBIC	Entropy	BLRT (p)
1	36549.259	36949.958	36676.866		
2	34867.911	35673.969	35124.609	0.911	0.0000
3	34124.132	35335.548	34509.920	0.967	0.0000
4	33641.826	35258.601	34156.705	0.918	0.0000

AIC, Akaike information criterion; BIC, Bayesian information criterion; aBIC, adjusted Bayesian information criterion; BLRT, bootstrap likelihood ratio test.

**Figure 1:**
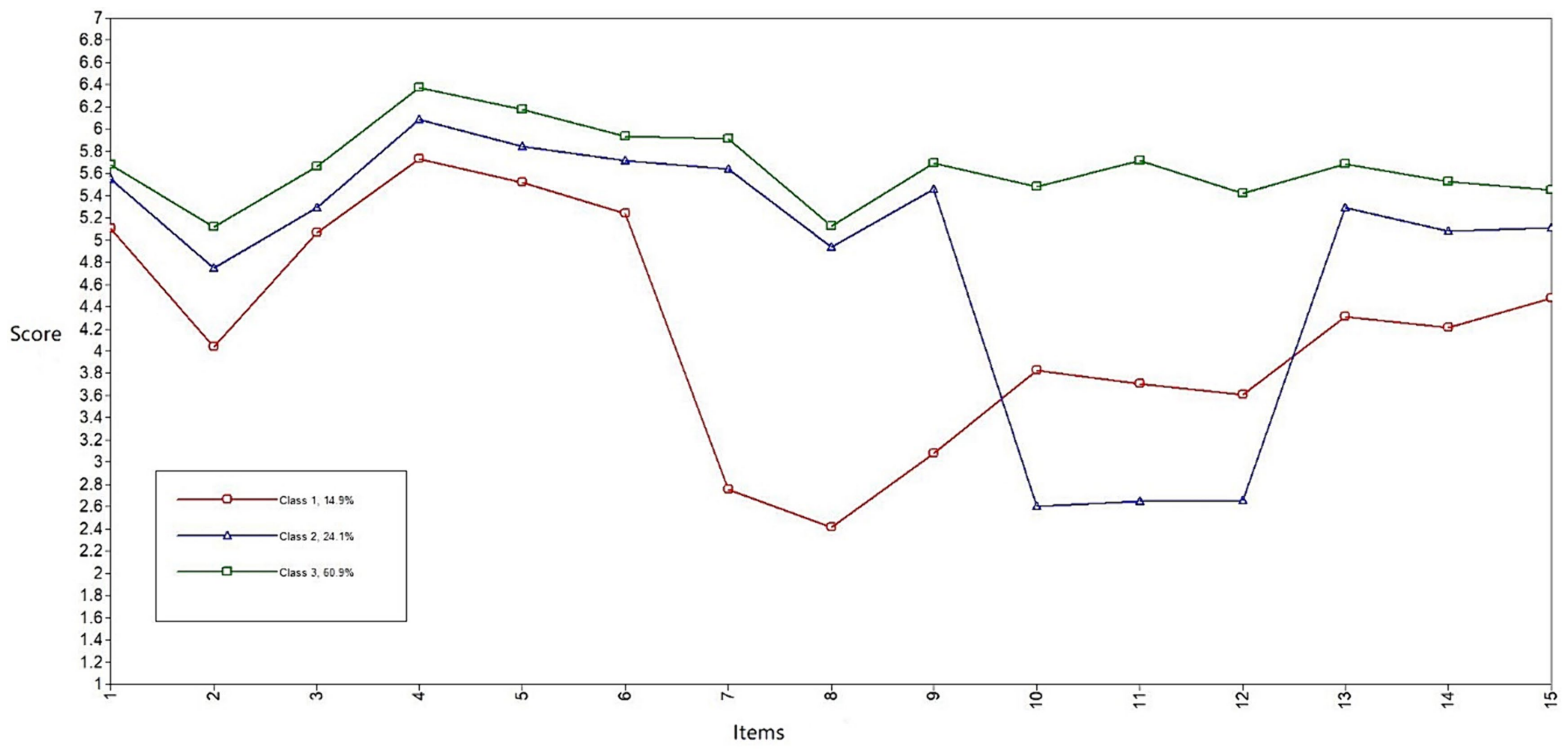
Scores of potential groups on each item of the Decent Work Scale.

The participants were divided into three groups based on the LPA results. Figure 1 shows the DWS scores for each group on the 15 items that assess five dimensions: security (questions 1–3), healthcare (4–6), salary (7–9), free time (10–12), and value fit (13–15). Based on the analysis of the results, the participants’ decent work can be classified into three latent types. The first group, named the ‘low pay group,’ had the lowest score in the salary dimension and low scores in all other items. The second group, named the ‘low free time group,’ had the lowest score in the free time dimension and middle-level scores in the other dimensions. Finally, the third group, named the ‘high decent work group,’ had the highest score on all items.

According to Table 2, the participants (*N* = 780) had an overall decent work score of 5.25 ± 0.72 (M ± SD), which was higher than the theoretical mean of the DWS (*M* = 4). Among them, the low pay group had the lowest decent work score, 4.21 ± 0.53 (M ± SD), which was lower than the overall sample mean but higher than the theoretical mean of the scale. The low free time group had the second lowest decent work score of 4.85 ± 0.46 (M ± SD), which was also higher than the theoretical mean of the scale but lower than the overall sample mean. The high decent work group had the highest decent work score at 5.66 ± 0.45 (M ± SD), which was higher than the overall sample mean.

**Table 2:** Decent work scores for potential groups.

Latent class	*N*	Decent work (M ± SD)
1. Low pay group	117	4.21 ± 0.53
2. Low free time group	188	4.85 ± 0.46
3. High decent work group	475	5.66 ± 0.45
4. Overall	780	5.25 ± 0.72

### 3.2 Differences in demographic variables of the latent profiles of decent work

Demographic variables were tested for differences in the latent profiles of decent work, and different testing methods were selected according to the variable types. Gender, marital status, education, and partner education were categorical variables, and the chi-square test was used to test for differences. The results are summarized in Table 3.

**Table 3:** Differences in latent profiles of decent work by gender, marital status, education, and partner education.

	Latent class			*χ^2^*	*p*
	Category 1	Category 2	Category 3		
Gender				3.175	0.204
Male	55 (17.3%)	69 (21.7%)	194 (61%)		
Female	62 (13.4%)	119 (25.8)	281 (60.8)		
Marital status				6.253	0.181
Unmarried	25 (19.7%)	34 (26.8%)	68 (53.5%)		
Married	91 (14.0%)	154 (23.7%)	406 (62.4%)		
Other	1 (50.0%)	0 (0%)	1 (50%)		
Education				11.705	0.165
High school and below	3 (50%)	1 (16.7%)	2 (33.3%)		
College	2 (12.5%)	5 (31.3%)	9 (56.3%)		
Undergraduate	75 (16.2%)	115 (24.8%)	273 (59.0%)		
Postgraduate	29 (12.3%)	58 (24.7%)	148 (63.0%)		
Ph.D.	8 (13.35%)	9 (15.0%)	43 (71.7%)		
Partner education				20.874	0.007
High school and below	3 (37.5%)	2 (25.0%)	3 (37.5%)		
College	16 (31.4%)	10 (19.6%)	25 (49.0%)		
Undergraduate	66 (15.5%)	102 (23.9%)	259 (60.7%)		
Postgraduate	23 (9.6%)	64 (26.8%)	152 (63.6%)		
Ph.D.	9 (16.4%)	10 (18.2%)	36 (65.5%)		

Age and socioeconomic status were continuous variables among the demographic variables measured, and their differences were tested using an analysis of variance. The results of the homogeneity of variance test showed that the variances of the groups were not homogeneous in terms of age or socioeconomic status (Levene’s *F*_age_ = 3.875, *p* = 0.021; Levene’s *F*_socioeconomic status_ = 4.328, *p* = 0.014). Welch’s test was used to determine whether there was a difference between groups. The results showed no significant difference in age among the groups, Welch’s *F*_age_ (df1, df2) = 2.968 (2, 251.140), *p* > 0.05. There were significant differences in socioeconomic status (Welch’s *F*_socioeconomic status_ (df1, df2) = 19.788 (2, 261.072), *p* < 0.001). The Games-Howell method was used for post-hoc multiple comparisons, and the results are presented in Tables 4, 5.

**Table 4:** Age differences in latent profiles of decent work.

Latent type	*N*	Age (M ± SD)	Welch		Games-Howell (p)	
			*F*	*p*	1	2
1	117	33.00 ± 6.82	2.968	0.053		
2	188	31.52 ± 6.70			0.155	
3	475	31.35 ± 5.37			0.042	0.947

**Table 5:** Socioeconomic status differences in latent profiles of decent work.

Latent type	*N*	Socioeconomic status (M ± SD)	Welch		Games-Howell (p)	
			*F*	*p*	1	2
1	117	5.35 ± 1.46	19.788	0.000		
2	188	5.68 ± 1.34			0.128	
3	475	6.15 ± 1.23			0.000	0.000

### 3.3 Difference in the positive effects of the latent profiles of decent work

An analysis of variance was conducted to examine the differences in work need satisfaction, job satisfaction, and life well-being among the latent profiles of decent work. The results of the homogeneity test indicated that the variance of each group was not equal in terms of work need satisfaction, job satisfaction, and life well-being (Levene’s *F*_work need satisfaction_ = 5.443, *p* = 0.004; Levene’s *F*_job satisfaction_ = 8.467, *p* < 0.05; Levene’s *F*_life well-being_ = 6.339, *p* = 0.002). Therefore, Welch’s test was used to examine the differences between profiles. The results showed significant differences in work need satisfaction, job satisfaction, and life well-being among the profiles: Welch’s *F*_work need satisfaction_ (df1, df2) = 61.620 (2, 256.992), *p* < 0.001; Welch’s *F*_job satisfaction_ (df1, df2) = 97.712 (2, 260.818), *p* < 0.001; Welch’s *F*_life well-being_ (df1, df2) = 68.596 (2, 256.937), *p* < 0.001. Post-hoc multiple comparisons using the Games-Howell method revealed significant differences between the groups, as presented in Tables 6–8.

**Table 6:** Differences in work need satisfaction among latent profiles of decent work.

Latent type	*N*	Work need satisfaction (M ± SD)	Welch		Games-Howell (p)	
			*F*	*p*	1	2
1	117	5.05 ± 0.67	61.620	0.000		
2	188	5.53 ± 0.64			0.000	
3	475	5.77 ± 0.55			0.000	0.000

**Table 7:** Differences in job satisfaction among latent profiles of decent work.

Latent type	*N*	Job satisfaction (M ± SD)	Welch		Games-Howell (p)	
			*F*	*p*	1	2
1	117	3.07 ± 0.67	97.712	0.000		
2	188	3.77 ± 0.55			0.000	
3	475	4.00 ± 0.54			0.000	0.000

**Table 8:** Differences in life well-being among latent profiles of decent work.

Latent type	*N*	Life well-being (M ± SD)	Welch		Games-Howell (p)	
			*F*	*p*	1	2
1	117	4.24 ± 1.07	68.596	0.000		
2	188	4.93 ± 1.03			0.000	
3	475	5.43 ± 0.89			0.000	0.000

## 4 Discussion

This study aimed to explore the latent profiles of decent work among Chinese employees using an individual-centered approach, providing a realistic and concrete description of their decent work conditions. The study utilized LPA and a five-dimensional DWS to identify groups with distinct decent work characteristics. Further, differences in demographic variables, such as gender, age, education, and socioeconomic status, as well as the positive effects of decent work, such as work need satisfaction, job satisfaction, and life well-being, were analyzed using various analytical methods.

The results of LPA showed that Chinese employees’ decent work could be divided into three types. The first type had the lowest scores on the three items of the salary dimension and lower scores on other items, which was named the ‘low pay group.’ The second type had the lowest score on the three items of the free time dimension, and the scores of the other items were at the middle level; this type was called the ‘low free time group.’ The third type involved the score on all items being the highest, known as the ‘high decent work group.’ This result was clearly at odds with the decent work research of Blustein et al. (3) and Kim et al. (4) on working adults in the United States. Blustein et al. (3) and Kim et al. (4) divided participants into five latent types. Taking the study of Kim et al. (4) as an example, an LPA of decent work in the white middle class with an annual income of approximately $50,000 was conducted. The participants were divided into five types: ordinary type (the scores of each dimension were close to the theoretical mean); low medical insurance type; high medical insurance type; under-decent type; decent type. The medical security dimension is a prominent indicator of the segmentation of profiles, and the medical security system in China differs significantly from that in the United States. China’s medical security system is dominated by social medical insurance, mainly for general income groups, which benefits several people. The financing method involves participating in insurance payments according to the law, and the service supply is mainly for social pricing fees and insurance payments, aiming to reduce the burden of medical expenses for residents. In the United States, the medical insurance system is based on commercial medical insurance, and benefits are concentrated in high-income groups. The financing method is mainly voluntary, and the service supply is shared at different prices (16). Therefore, the impact of healthcare indicators on the latent profile of decent work among Chinese workers is distinct from that observed in the U.S. context. The difference in the results of the two studies also suggests the need for decent work research in the Chinese context. As a special concept, the influence of social background factors, such as socio-political economy, historical culture, and mainstream values, should be considered in decent work research.

In this study, the total score of decent work for all participants (5.25 ± 0.72) was higher than the theoretical mean value of the scale (*M* = 4), indicating that the level of decent work among Chinese employees is relatively high. The low pay group had the lowest average score for decent work, indicating that jobs with a low level of decent work mainly reflected a low level of pay. The low free time group had the second lowest average score, indicating that participants were generally satisfied with the decent component of their work but dissatisfied with the work occupying their personal time and not having sufficient time to rest. Conversely, the high decent work group had the highest average score for all five dimensions. Notably, the scores of all three groups fluctuated downward for questions 2 and 8, indicating that all participants felt pressure from interpersonal aspects at work, regardless of the group to which they belonged. In terms of income, participants were satisfied with their income when not compared to others. Nevertheless, upon comparing to others, they felt their income was not ideal. According to the ‘theory of social comparison’ (33), people enhance the cognition of their situation through comparison, which also leads to psychological gaps.

The low pay group felt their income was not ‘decent,’ and their scores on free time were lower (although higher than those of the low free time group), and their values aligned with organizational values were the lowest among the three groups. According to Maslow’s (34) hierarchy of needs theory, material needs are related to an individual’s survival. Insufficient material needs can directly endanger an individual’s life and inhibit the satisfaction of their growth needs. Growth needs are much more complex than material needs, as they involve favorable external conditions, such as economic and social conditions (35). Salary is the main source of labor consumption goods. Individuals exchange labor income for life necessities and other consumer goods, which belong to the category of typical material needs. The low pay group has a lower income, and their material needs are not met. Therefore, they pay less attention to safe environments, medical security, and organizational value fit at work. In fact, they must give up more freedom and rest time in exchange for a higher salary, which is reflected in their low scores in the free time dimension.

It is suggested that organizations should pay more attention to employees’ actual work experiences and life needs while focusing on their performance to ensure reasonable rest time. Additionally, organizations should adopt various methods and strategies to convey their values to employees and enhance the fit between employee and organizational values. Individual employees should focus on continuous learning and self-improvement, enhancing their business knowledge and capabilities, and striving for more opportunities to obtain decent jobs.

The decent work characteristics of the participants in the second latent category (i.e., the low free time group) were more clearly defined. The participants in this group provided relatively positive evaluations of the four decent work components of safe environment, healthcare, appropriate salary, and value fit, which were in the middle of the three potential categories. However, the score for the decent component of free time was the lowest among the three potential categories. According to the resource conservation theory (36), individuals have limited resources, and when they face stressful situations, they first consume secondary resources to avoid the loss of priority resources. When dealing with stressful work situations, some employees choose to sacrifice their personal time to complete tasks without punishment. Nevertheless, according to the recovery theory, employees need sufficient time to recover after work physically and mentally. If recovery is insufficient, the neurobiological system will remain activated and will not return to a state of homeostasis (37). Employees in their suboptimal form will have to make extra efforts to cope with the demands of the next job, potentially leading to further long-term fatigue (38). This may also provide an explanation for other scores in the low free time group that were below those in the high decent work group. Therefore, it is recommended that organizations provide additional guidance and learning opportunities to these employees. Such support could help them improve their time management skills, enhance their business capabilities and working methods, and ultimately increase their chances of obtaining high-quality decent jobs.

In the overall sample, the high decent work group comprised the highest proportion (60.9%) among participants, indicating that most employees had a relatively high level of employment quality and work experience. In comparison, Blustein et al. (3) and Kim et al. (4) reported proportions of the decent work group to be 11.2 and 31.8%, respectively. While these differences are notable, it’s important to interpret them cautiously. Given the similar proportions of the middle class in the samples of these studies and our research, these findings might suggest potential differences in perceived employment quality and work experiences between our Chinese sample and the American samples in the cited studies. However, due to our limited sample size and potential sampling biases, we cannot conclusively generalize these results to all Chinese employees or make definitive cross-cultural comparisons. Further research with larger, more representative samples would be needed to confirm these preliminary observations.

These results have important implications for the construction and measurement of decent work in previous studies. As mentioned earlier, some studies (2, 15) have assumed population homogeneity in the sample and treated decent work as a co-increase or co-decrease or an all-or-nothing single-dimensional concept. However, the findings of this study suggest that the components of decent work do not always co-occur, especially regarding pay and free time. This means that not every job has all the necessary components for decent work. These results also suggest that while decent work may be appropriate to measure as a whole in some circumstances, it may be more appropriate to consider subscales of decent work that may be disjoint; these subscales should be carefully selected based on actual circumstances and subjects.

Differences in demographic variables and the positive impact of decent work across groups were analyzed using various analytical methods. First, the differences in gender, age, marital status, education, partner education, and socioeconomic status among the three potential types were examined using a chi-square test. The results showed no significant differences in gender, marital status, or educational background among the three potential types. Nonetheless, there were significant differences in the partners’ educational backgrounds and socioeconomic status. Specifically, the higher the educational background of the partner, the greater the proportion of the ‘high decent work group.’ The higher the socioeconomic status, the greater the proportion of the ‘high decent work group.’

There were no significant gender differences among the three potential types (*χ^2^* = 3.175, *p* = 0.204), which is consistent with Blustein et al. (3) and other decent work studies based on the Psychology of Working theory (39) but not with the findings of Kalleberg (40) and Kim et al. (4). Kalleberg (40) shows that women are disadvantaged in employment and are more likely to obtain unsafe and precarious jobs. Kim et al. (4) show that women comprise a higher proportion in the under-decent group (78.6%), low medical insurance group (77.8%), and average group (76.8%). Although this study is inconsistent with similar studies in terms of gender differences, it does not deny the impact of gender in the workplace. While gender is not prominent in predicting decent work, future research could consider the relationship between gender and other work or workplace factors such as income gaps, promotion ceilings, and workplace sexual harassment.

There were no significant differences in age or education among the three latent types, which is inconsistent with the results of previous studies. Referring to similar studies, Blustein et al. (3) and Kim et al. (4) reported the effect of age and education on decent work—the older the age, the smaller the proportion of participants in the high decent work group, and the higher the education, the greater the proportion of participants in the high decent work group. In terms of age, this may be due to inconsistent results occurring from differences in workplace cultures between China and the United States. The Oriental workplace culture represented by China respects seniority, where people acquire the same type of position, and the longer they work, the more experience they have. The social culture of respecting older adults may also play a role in promoting seniority worship (41). In terms of educational level, some participants may have developed the perception that their educational level was higher than their job requirements (i.e., excess qualifications), and they did not have the opportunity to display their talents fully. Their efforts were not properly rewarded, which, in turn, led to emotions such as self-reward and self-compassion (42). This may have reduced the participants’ perceptions of the decent elements of their work. Additionally, some highly educated subjects may be overly relied upon by their work units and appear to have role overload, role ambiguity, and so on, leading to excessive occupation of personal time and space, psychological exhaustion, depersonalisation, and other burnout states (43). In turn, these aspects decrease individuals’ perceptions of the decent component of work. Consequently, some highly educated participants were also distributed in the low pay and low free time groups in this study.

The three potential types differed in terms of partner education and socioeconomic status. The higher the partner’s education, the greater the proportion of the ‘high decent work group.’ There are two possible explanations for this finding. First, participants with high decent job characteristics may have more advantages in terms of marriage and love. They have safe and stable jobs, appropriate remuneration, and sound medical insurance. Further, they are highly aligned with organizational values and have broad job prospects. Thus, they are more likely to attract partners with high-quality characteristics (such as a high degree of education). According to an old saying, ‘only when the phoenix tree is planted can the golden phoenix be attracted.’ Therefore, participants with high decent work characteristics are more likely to meet a partner with superior mate characteristics. Another explanation is that highly educated partners usually have higher cognitive levels and work-related abilities or skills; hence, they can provide more support for their significant others, especially in terms of family-work facilitation (44). According to the spillover theory, people bring the knowledge, experiences, emotions, skills, and behaviors built up at work into the home domain and vice versa (45). Highly educated couples can share their knowledge and experience, helping each other improve their business skills, increase their interpersonal skills in the workplace, or provide more social support for their partners in other aspects. These aspects help each other cope more effectively with work challenges, gain efficiency and effectiveness, enhance positive evaluations of work, and improve the level of decent work. Individuals with high socioeconomic status have more resources and support and greater opportunities and possibilities to obtain decent work (1). Thus, their economic status will also be rated higher than those with low levels of decent work. These two explanations are consistent with previous findings on decent work (39).

Furthermore, Welch’s test was used to determine the difference in the positive impact of decent work among the three potential types, and the results showed that there were significant differences among the groups in work need satisfaction, job satisfaction, and life well-being. This finding is consistent with the Psychology of Working theory (39) and previous studies on decent work (3, 4). These results show the actual work experience of Chinese employees more clearly and provide supporting evidence from China for the Psychology of Working theory.

Our findings showed that the high decent work group also had the highest percentage of high scores in work need satisfaction, while the low pay group had the lowest work need satisfaction, consistent with the theoretical expectations of Psychology of Working framework (1, 26). These findings on job satisfaction and well-being are consistent with previous research and theories (10, 46). Participants with high decent work were more likely to report high job satisfaction and well-being, supporting the basic framework of the Psychology of Working theory (1).

This study used Chinese employees as a sample and applied an individual-centered LPA method to conduct a more realistic investigation of their decent work characteristics. This approach supports the Psychology of Working theory and expands its cross-cultural application scope.

While some of our findings may be specific to the Chinese context, others potentially offer generalizable insights into decent work across cultures. The emergence of a low pay profile aligns with global concerns about income inequality and working poverty (12), suggesting that inadequate compensation remains a critical issue in defining decent work across contexts. Similarly, the identification of a low free time profile reflects growing global concerns about work-life balance and the “always-on” work culture facilitated by technology (47).

However, the absence of a healthcare-focused profile, which was prominent in U.S. studies (4), may be specific to the Chinese context, reflecting the country’s near-universal healthcare coverage (48). This highlights the importance of considering national policies and systems when studying decent work across cultures.

The strong association between the high decent work profile and positive outcomes (work need satisfaction, job satisfaction, and life well-being) appears to be consistent across cultures, supporting the universal relevance of decent work for employee well-being as proposed by the Psychology of Working Theory (1). Besides, the three latent types showed significant differences in demographic and outcome variables, suggesting that decent work is not an all-or-nothing construct and that its intrinsic components should be flexibly combined based on the research context and purpose.

## 5 Contributions

There are three key contributions of this study. First, we provide empirical evidence for the heterogeneity of decent work experiences within the Chinese workforce, challenging the notion of decent work as a unidimensional construct. Second, we demonstrate the significant associations between decent work profiles and important outcomes such as work need satisfaction, job satisfaction, and life well-being, reinforcing the critical role of decent work in employee well-being. Third, we highlight the unique characteristics of decent work in the Chinese context, particularly the absence of a healthcare-focused profile and the prominence of free time as a distinguishing factor.

These findings have important implications for theory, research, and practice. Theoretically, they support the core tenets of the Psychology of Working Theory while also suggesting the need for cultural adaptations. For researchers, our results underscore the importance of using person-centered approaches and considering cultural context in the study of decent work. Practically, our findings can inform organizational policies and interventions aimed at promoting decent work, particularly in addressing issues of compensation and work-life balance.

## 6 Limitations and prospects

This study has some limitations. Although efforts were made to recruit as many participants as possible, the study’s sample size was limited and may not be representative of the entire working population in China, particularly because of the COVID-19 pandemic. Furthermore, all participants in this study were working adults, most of whom worked in state-owned enterprises and government agencies and had a high level of education. Approximately 97.1% of the participants had a bachelor’s degree. An important limitation of our sampling method is that it may have excluded very low-income workers or those facing difficulties in accessing or understanding the questionnaire due to limited literacy or technological access. This potential exclusion of marginalized workers is a concern in decent work research, as these populations are often most vulnerable to indecent work conditions. Future studies should strive to include more diverse samples and develop methodologies to reach and accurately assess the experiences of workers across all socioeconomic levels and literacy backgrounds. Therefore, caution should be exercised when generalizing these findings to other populations, particularly to those in informal employment, migrant workers, or those with lower educational attainment. Future studies should aim to recruit more diverse samples that better represent the full spectrum of the Chinese workforce. Moreover, in the difference test, only demographic and outcome variables were examined; other predictors in the framework of the Psychology of Working theory were not investigated. Future research should explore the longitudinal dynamics of decent work profiles, investigate potential mediators and moderators of the relationship between decent work and outcomes, and conduct cross-cultural comparisons to further elucidate the universal and context-specific aspects of decent work. Additionally, examining decent work in the growing gig economy and among migrant workers in China could provide valuable insights into the evolving nature of work in the country. Researchers should also consider developing and validating methods to assess decent work among populations with varying levels of literacy and access to technology, ensuring that future studies can capture a more comprehensive picture of decent work across all segments of society.

## 7 Conclusion

This study provides novel insights into the nature and implications of decent work in the Chinese context, contributing to the growing body of international research on this crucial concept. Our findings reveal three distinct profiles of decent work among Chinese employees: low pay, low free time, and high decent work. These profiles demonstrate both similarities and differences compared to those identified in Western contexts, highlighting the importance of cross-cultural research in this area.

## Data Availability

The raw data supporting the conclusions of this article will be made available by the authors, without undue reservation.
